# Interstrain Variability of Human Vaginal *Lactobacillus crispatus* for Metabolism of Biogenic Amines and Antimicrobial Activity against Urogenital Pathogens

**DOI:** 10.3390/molecules26154538

**Published:** 2021-07-27

**Authors:** Scarlett Puebla-Barragan, Emiley Watson, Charlotte van der Veer, John A. Chmiel, Charles Carr, Jeremy P. Burton, Mark Sumarah, Remco Kort, Gregor Reid

**Affiliations:** 1 Canadian Centre for Human Microbiome and Probiotics, Lawson Health Research Institute, 268 Grosvenor Street, London, ON N6A 4V2, Canada; spueblab@uwo.ca (S.P.-B.); ewatso26@uwo.ca (E.W.); jchmiel4@uwo.ca (J.A.C.); charlie.carr@mail.utoronto.ca (C.C.); Jeremy.Burton@lawsonresearch.com (J.P.B.); 2 Departments of Microbiology and Immunology, and Surgery, Western University, London, ON N6A 4V2, Canada; 3 Department of Infectious Diseases, Public Health Service (GGD), Nieuwe Achtergracht 100, 1018 WT Amsterdam, The Netherlands; cvanderveer@mlw.mw; 4 Agriculture and Agri-Food Canada, London, ON N5V 4T3, Canada; mark.sumarah@canada.ca; 5 Department of Molecular Cell Biology, Faculty of Science, O2 Lab Building, Vrije Universiteit Amsterdam, De Boelelaan 1108, 1081 HZ Amsterdam, The Netherlands; r.kort@vu.nl; 6 ARTIS-Micropia, Plantage Kerklaan 38-40, 1018 CZ Amsterdam, The Netherlands

**Keywords:** *Lactobacillus crispatus*, vaginal microbiome, probiotics, metabolomics

## Abstract

*Lactobacillus crispatus* is the dominant species in the vagina of many women. With the potential for strains of this species to be used as a probiotic to help prevent and treat dysbiosis, we investigated isolates from vaginal swabs with *Lactobacillus*-dominated and a dysbiotic microbiota. A comparative genome analysis led to the identification of metabolic pathways for synthesis and degradation of three major biogenic amines in most strains. However, targeted metabolomic analysis of the production and degradation of biogenic amines showed that certain strains have either the ability to produce or to degrade these compounds. Notably, six strains produced cadaverine, one produced putrescine, and two produced tyramine. These biogenic amines are known to raise vaginal pH, cause malodour, and make the environment more favourable to vaginal pathogens. In vitro experiments confirmed that strains isolated from women with a dysbiotic vaginal microbiota have higher antimicrobial effects against the common urogenital pathogens *Escherichia coli* and *Enterococcus faecium*. The results indicate that not all *L. crispatus* vaginal strains appear suitable for probiotic application and the basis for selection should not be only the overall composition of the vaginal microbiota of the host from which they came, but specific biochemical and genetic traits.

## 1. Introduction

As the dominant organism in the vagina of many healthy women, *Lactobacillus crispatus* is thought to be an important contributor to reproductive health [1]. For this reason, the species has been postulated to be an excellent candidate for probiotic use to restore and maintain vaginal health [2]. Given the high incidence of conditions that result from vaginal dysbiosis [3,4,5], including urinary tract infection (UTI) and bacterial vaginosis (BV), as well as an increased risk of sexually transmitted diseases and preterm labour [5], the vaginal administration of one strain of *Lactobacillus crispatus* has been pilot tested to improve women’s health [6]. In general, the ability of lactobacilli to adhere to vaginal epithelial cells and interfere with the adhesion of pathogenic microbes, are believed to be important mechanistic contributors [7], as well as a broad-spectrum inhibitory activity against a range of Gram-positive and Gram-negative bacteria [8]. This is in part due to the production of lactic acid, but also other anti-microbial molecules, including bacteriocins, may be involved.

Another relevant marker of female urogenital health is the presence or absence of large amounts of biogenic amines such as putrescine, cadaverine, and tyramine [9]. These compounds, commonly produced by urogenital pathogens, are elevated in patients with BV [4] and UTI [10] and are known causes of malodour. Beyond that, large amounts of these compounds increase the risk of vaginal dysbiosis [11]. Therefore, a high abundance of strains that reduce the amounts of biogenic amines are desirable in the vaginal environment. 

However, it is not clear if these properties and the prevalence of *L. crispatus* species are sufficient to select candidate probiotic strains. As bacterial metabolites play a role in host health and disease, the present study was designed to examine the by-products of strains of *L. crispatus* isolated from healthy women and those with dysbiosis [12]. Liquid chromatography-mass spectrometry (LC-MS) was used, given its ability to differentiate by-products of vaginal microbiota strains [4]. 

## 2. Results

### 2.1. Untargeted Metabolomics and Functional Genomics

**Figure 1:**
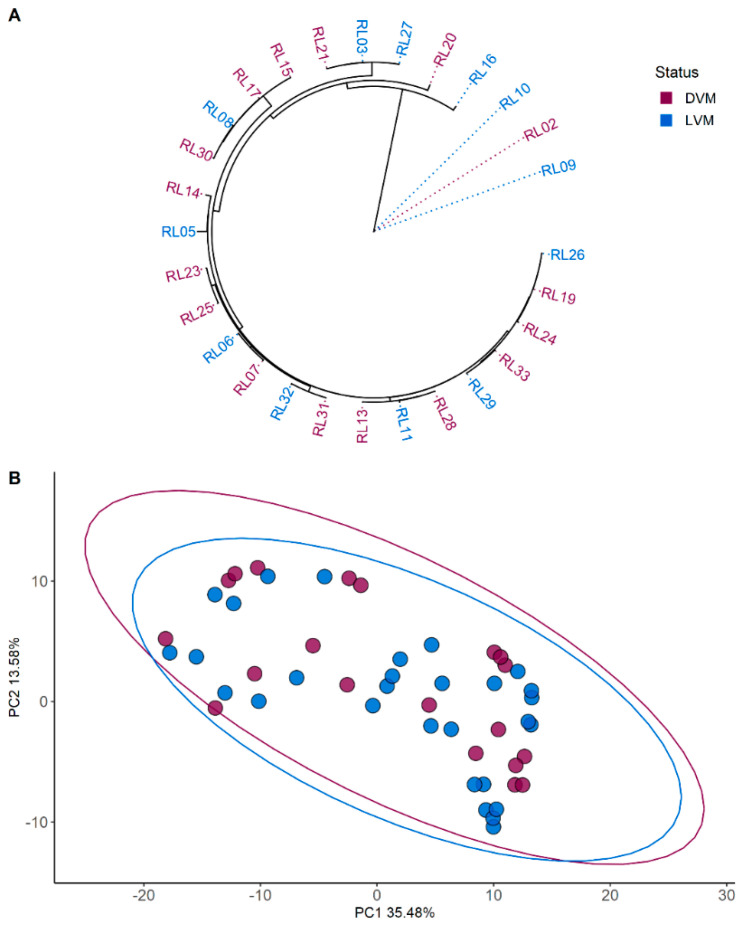
Phylogenetic and metabolomic clustering analyses. (**A**) Core gene phylogenetic tree based on 28 strain sequences. (**B**) Untargeted metabolomics based on 18 strains. Ellipses represent the 95% confidence intervals. Colour coding represents grouping. Burgundy represents strains isolated from dysbiotic vaginal microbiota (DVM) and blue represents strains isolated from *Lactobacillus* dominated vaginal microbiota (LVM).

PCA analysis of the metabolites identified through LC-MS did not find any clustering between the *L. crispatus* strains as shown in Figure 1. Similarly, neither the phylogenetic nor the functional analyses revealed any specific grouping according microbiota status (Figure 1A and Figure S1). 

### 2.2. Targeted Metabolomics

**Figure 2:**
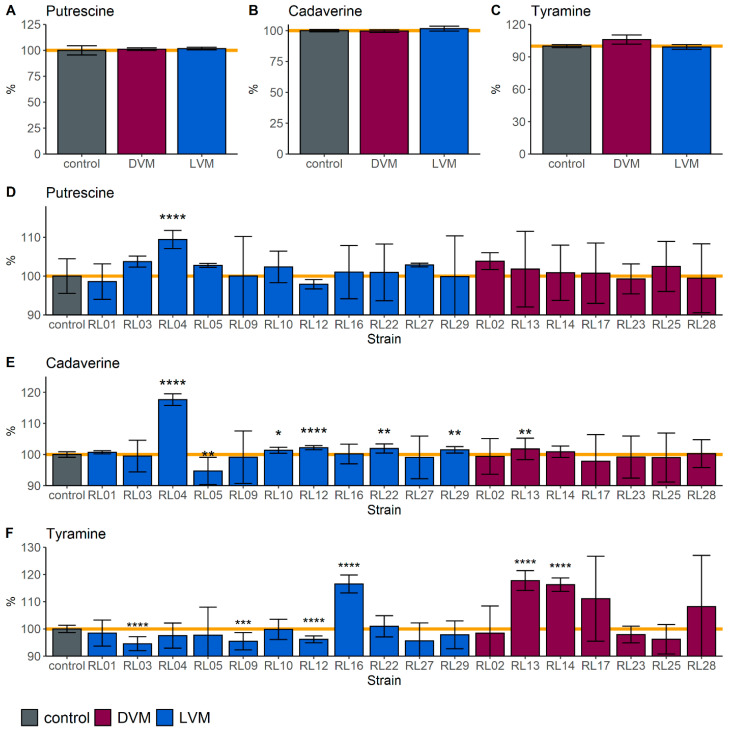
Production and degradation of biogenic amines. Semi-quantitative metabolomics analysis of the differences in putrescine (**A**,**D**), cadaverine (**B**,**E**), and tyramine (**C**,**F**), between the control (VDMP media only) and spent media by microbiota status (**A**–**C**) and by strain (**D**–**F**). Burgundy represents strains isolated from dysbiotic vaginal microbiota (DVM) and blue represents strains isolated from *Lactobacillus* dominated vaginal microbiota (LVM). Data are represented as the means of 3 independent experiments (* *p* < 0.05, ** *p* < 0.01, *** *p* < 0.001, **** *p* < 0.0001).

Between isolated strains from *Lactobacillus*-dominated and dysbiotic vaginal microbiota groups, no significant differences were identified in the amounts of putrescine, cadaverine, and tyramine (Figure 2). Semi-quantitative analysis was based on the areas under the curve of the features corresponding to the amines already present in the media, or those produced after incubation. 

### 2.3. Biogenic Amines Pathway Analysis

**Figure 3:**
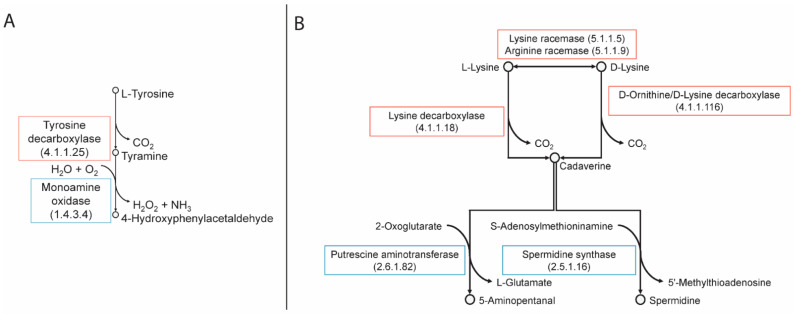
Metabolic pathways for the synthesis and degradation of tyramine (**A**) and cadaverine (**B**).

**Figure 4:**
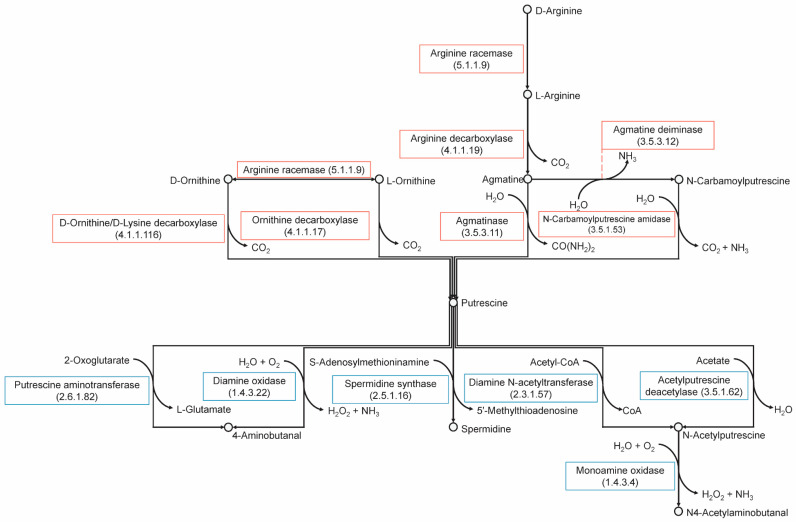
Metabolic pathways for the synthesis and degradation of putrescine.

The analysis of the biosynthetic and biocatalytic pathways for putrescine, tyramine, and cadaverine (Figure 3 and Figure 4) revealed that all strains possess the gene that encodes for the enzyme lysine racemase, which catalyzes the bidirectional reaction from L-lysine to D-lysine. The gene encoding for lysine decarboxylase was absent from all strains; this enzyme is responsible for the decarboxylation of L-lysine. However, all strains appear to have the gene that encodes for the enzyme D-ornithine/D-lysine which decarboxylates D-lysine into cadaverine and D-ornithine into putrescine. Only six strains have the encoding sequence for agmatine deiminase (RL03, RL14, RL16, RL20, RL31, RL32), however none have the gene encoding N-carbamoylputrescine amidase, which is required in addition to produce putrescine from agmatine. All strains have the genes that encode for arginine decarboxylase and arginine racemase which convert L- and D-arginine into putrescine, respectively. Arginine racemase also catalyzes the two-way reaction between L- and D-ornithine. All strains also possess the gene for ornithine decarboxylase which synthesizes putrescine from L-ornithine. Only strain 20 has the gene encoding for tyrosine decarboxylase, which converts L-tyrosine into tyramine.

**Figure 5:**
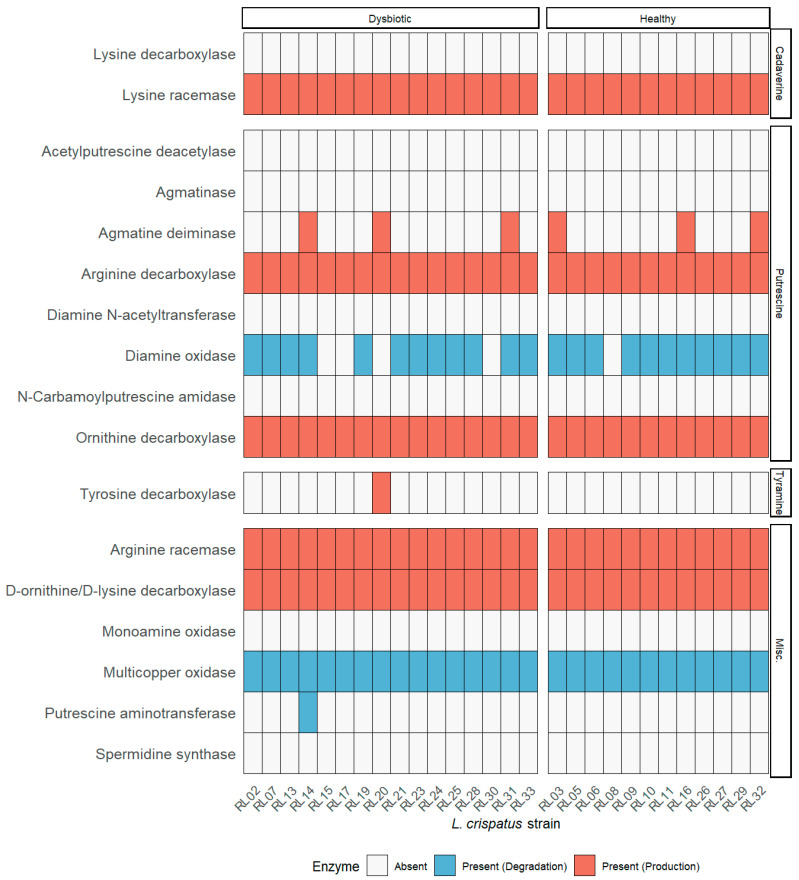
Key enzymes in the metabolism of biogenic amines. Summary of the presence or absence of genes encoding for the enzymes involved in the biosynthesis (red) or degradation (blue) of biogenic amines. All available genomes were used for this analysis.

Of the degrading enzymes, all strains except RL09, RL15, RL17, RL20, and RL30 have the gene for the enzyme diamine oxidase, which converts putrescine into 4-aminobutanal. Strain 14 also has the gene for putrescine aminotransferase, which also converts putrescine into 4-aminobutanal. Additionally, all strains have genes encoding multicopper oxidases. A summary of these findings can be found in Figure 5.

### 2.4. Antibacterial Activity

**Figure 6:**
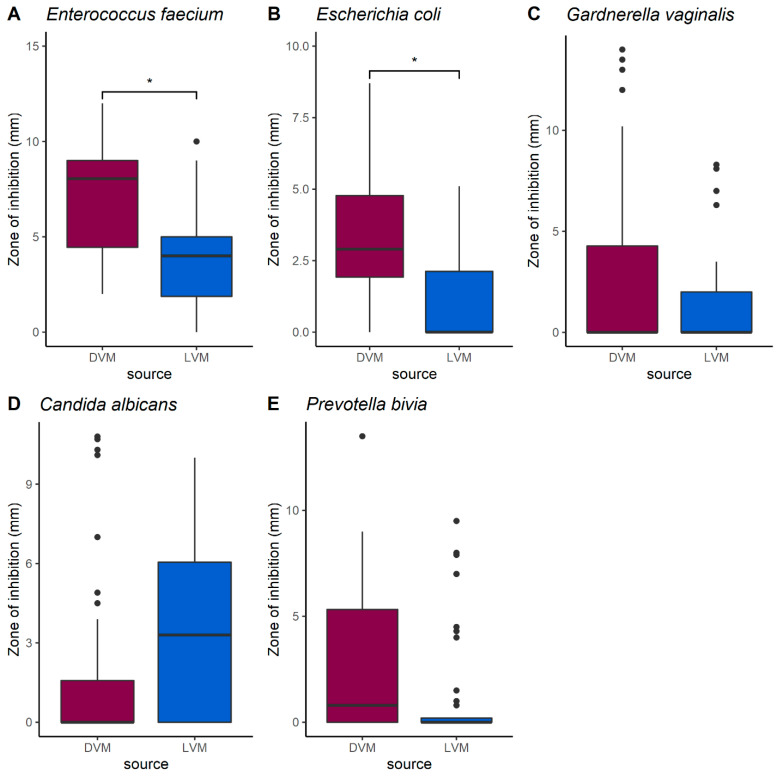
Differences between the antimicrobial capacity of *L. crispatus* strains isolated from dysbiotic and *Lactobacillus* dominated vaginal environments. Against (**A**) *Enterococcus faecium*, (**B**) *Escherichia coli*, (**C**) *Gardnerella vaginalis*, (**D**) *Candida albicans*, and (**E**) *Prevotella bivia.* Burgundy represents strains isolated from dysbiotic vaginal microbiota (DVM; *n* = 8) and blue represents strains isolated from *Lactobacillus* dominated vaginal microbiota (LVM; *n* = 11). A linear mixed effects model to control for individual strain effects was used to evaluate differences between the groups (* *p* < 0.05).

Agar well diffusion assays were conducted using 19 of the *L. crispatus* strains, against *E. faecium*, *E. coli*, *G. vaginalis*, *P. bivia*, and *C. albicans* as indicator strains. Results showed that strains isolated from dysbiotic patients were more effective in inhibiting *E. faecium* and *E. coli* than those isolated from healthy patients (Figure 6). No significant differences were observed in the inhibition of *G. vaginalis*, *C. albicans*, and *P. bivia*.

The supernatants and treatments with heat and trypsin showed no inhibition of *E. faecium* (Supplementary Figure S3).

## 3. Discussion 

Although the *L. crispatus* species has been proposed as an excellent candidate for probiotic use to restore and maintain vaginal health because of its preponderance in healthy women, the present study showed that not every strain necessarily has the ideal properties. Untargeted metabolomic analysis of 18 vaginal strains of *Lactobacillus crispatus* did not show any distinguishable groupings fitting healthy versus dysbiotic origins. Similarly, genomic and functional analyses of all available genomes did not reveal any specific pattern to suggest that the health status of the source host plays a significant role in the specific characteristics of the strains. Altogether, the results showed that all the strains are highly similar both in terms of genes and metabolism.

However, when a more specifically focused targeted metabolomic analysis was performed of the production and degradation of biogenic amines it showed that strains have either the ability to produce or to degrade these compounds. In addition, the evaluation of specific amino acid sequences and metabolic pathways showed that all strains have the genetic potential to produce and to degrade cadaverine and putrescine, while only strain RL20 can produce tyrosine decarboxylase which produces tyramine. Nonetheless, data obtained in vitro showed that other two strains (i.e., RL16 and RL14) can produce tyramine when grown in conditions that simulate the vaginal environment (unfortunately strain RL20 was not available for this analysis). These findings have implications for products using *L. crispatus* ostensibly for vaginal health, as potentially some of these strains may actually produce malodorous compounds. These results suggest that targeted approaches are better suited for the identification of probiotic candidates than those that provide a global overview.

In a study of another lactic acid bacterium, *Enterococcus faecium* E17, the tyrosine decarboxylation pathway was found to be a key survival mechanism in low acid and nutrient-depleted conditions [13]. Within enterococci, the functioning of this pathway also contributes to the binding and immunomodulation of enterocytes. Another species, *Enterococcus durans* IPLA655, uses this ability as a survival and colonization mechanism that enhances adhesion to the intestinal epithelium [14]. The capacity of *L. crispatus* to utilize this pathway could be a method of removing the tyrosine for use by enterococci, similar to the iron sequestering abilities of *L. crispatus* during menstruation [15].

The production of biogenic amines, within the vagina is linked to an increase in pH which promotes colonization by BV-associated bacteria. Although all strains showed the genetic potential to produce cadaverine and putrescine, only six strains (i.e., RL04, RL10, RL12, RL13, RL22, and RL29) produced cadaverine in vitro and one (i.e., RL04) produced putrescine. This could be a result of an adaptation of these strains to conditions of higher pH, and under stressful in vitro conditions they reacted by raising the pH. Unfortunately, this makes the environment more favourable to pathogens, plus these compounds emit malodour. Such characteristics are not ideal for vaginal probiotics.

One strain showed in vitro capacity to degrade cadaverine (RL05), and six could reduce the amount of tyramine (RL03, RL09, RL12, RL13, RL14, and RL26). Based on our analysis of metabolic pathways, degradation of putrescine is most likely being converted to 4-aminobutanal through diamine oxidase, while tyramine is most likely to be degraded by a multicopper oxidase [16,17]. Interestingly, there are strains, such as RL01 and RL04 that both produce and degrade biogenic amines. This suggests an adaptation ability of the species depending on what compounds are in the surroundings. The required enzymes for degrading activities are present in the genomes, so it might require certain conditions and larger concentrations of amines in the media to activate them. This capacity makes them good probiotic candidates. A *L. crispatus* based probiotic that could degrade malodorous compounds efficiently would be a welcome addition to the current treatments of conditions such as BV.

Admittedly, only one strain was isolated from each host [12], which makes it possible that several *L. crispatus* strains co-exist in the vaginal environment creating a symbiotic relationship where some produce biogenic amines and others can degrade them. More studies are required to better characterize this relationship. Furthermore, not all strains were tested in vitro nor sequenced because they were not viable under the applied lab conditions. The vaginal environment is complex and simulating it adequately enough for all strains to grow and thrive in vitro has not yet been accomplished. Research on the specific growth requirements for reproducible growth of these strains is on-going.

Antimicrobial activity was significantly higher in the strains isolated from dysbiotic patients, suggesting adaptation due to be sourced from more competitive environments. This inhibitory activity ceased following treatment with the protease trypsin, and heating of the supernatants to 85 °C for 45 min, indicating that the observed antimicrobial properties resulted from a heat-labile protein. This suggests a potential bacteriocin, compounds regarded as being important defence factors for lactobacilli [18]. These antimicrobial proteins typically have a very narrow spectrum, usually only inhibiting closely related species [19]. However, those produced by *Lactobacillus* spp. are known to have a much broader spectrum of activity, including against Gram-negative bacteria [20]. Additional studies are required to express and characterize the putative antimicrobial peptides that are causing this activity.

Strain *L. crispatus* CTV-05 is already being developed as an intravaginal probiotic, but it only appears to be effective if the subject is devoid of indigenous *L. crispatus* [21]. This might suggest competition for receptor sites or some other quorum sensing effect that limits foreign strain colonization, but these mechanisms have not been explored to date and its ability to produce or degrade biogenic amines has not been reported.

In summary, the findings from the present study suggests that metabolic readouts along with a strain’s genomic capacity should be part of the characterization of candidate probiotics. There is a strong need to improve women’s health. The option of using probiotic *L. crispatus* strains to this end, is worthy of further study, but specific, well-documented characteristics must guide the selection.

## 4. Materials and Methods

### 4.1. Bacterial Strains Used in This Study

The strains, listed in Table 1, were collected as outlined by van der Veer et al. [12] with all material approved by each subject with written consent and prior approval obtained from the Institutional Review Board (IRB).

In brief, swabs were obtained from the Sexually Transmitted Infections clinic in Amsterdam, the Netherlands. Subjects RL01, RL03, RL04, RL05, RL06, RL08, RL09, RL10, RL11, RL12, RL16, RL21, RL22, RL26, RL27, and RL32 were healthy women whose vaginal swabs were dominated by lactobacilli (LVM) as determined by a Nugent score 0–3; the other subjects had a dysbiotic vaginal microbiota (DVM) with Nugent score 7–10. The swabs were plated on modified Trypticase Soy Agar and incubated anaerobically at 37 °C for 24 h. Following this, single colonies underwent 16S sequencing for identification purposes. The strains were then cryopreserved at −80 °C in vaginally defined medium plus peptone (VDMP) [22]. A total of 28 strains were genome sequenced using Illumina MiSeq to generate FASTQ workflow. The genomes were assembled, reordered, and annotated and deposited at DDBJ/ENA/GenBank (Table S1). 

The 28 strains along with an additional four strains (RL01, RL04, RL12, RL22) were brought to Canada for antimicrobial analysis but unfortunately 13 did not survive. (Table S1). One strain, RL33, did not then grow sufficiently well for metabolomic analysis. 

For antimicrobial testing, *Enterococcus faecalis* ATCC 19433 [23], an oral isolate of *Lactobacillus helveticus* [24], and a beer isolate of *Pediococcus pentosaceus* [25], were used as positive controls for the production of bacteriocins. Strains from uropathogenic species: *Enterococcus faecium* ATCC 19,434 [26], *Escherichia coli* UTI 89 [27], *Enterococcus faecalis* ATCC 19,433 [28], *Gardnerella vaginalis* ATCC 14,018 [29], *Prevotella bivia* ATCC 29,303 [29], and *Candida albicans* TIMM 1768 [30], were used as indicator strains. 

### 4.2. Phylogenetic and Functional Genomics Analyses

To determine if there was a correlation between the health status of the source of the strains and their genomic profiles, available *L.* *crispatus* genomic assemblies from BioProject PRJNA390079 were downloaded (NCBI; April 2021). All genomes were assessed for quality using QUAST v5.0.2 [31] and completeness using CheckM v1.1.3 [32]. Genomic assemblies with N50 < 10 kb and completeness of less than 95% were excluded from further analysis. All 28 genomic assemblies passed the quality control and were annotated using Prokka v1.14.5 [33]. The --gram pos and --mincontiglen 200 (bp) were specified. The pangenome was determined using Roary v3.13.0 with the assumptions that a core gene is defined as gene found in all but one of the isolates (>95%) and a minimum percentage identify for blastp of 99% [34]. The core gene phylogenetic tree was constructed using the core gene alignment from Roary in RAxML v8.2.12 [35] with the flags, -f a, -# autoMRE, and -m GTRGAMMA, and visualized using the R package ggtree v2.4.1 [36]. Functional capacity of the genomes was analyzed using eggNOG v5.0 and eggNOG-mapper v2 using the Lactobacillaceae database [37,38]. Distance matrices for functional category abundance was calculated using the R package vegan v2.5-7 [39] following an established method [40,41], and plotted using the R package ggplot2 v3.3.3 [42].

### 4.3. LC-MS Protocol 

Three individual colonies were selected from each strain as independent biological replicates. Next, they were grown anaerobically for 24 h in VDMP media at 37 °C. Following the addition of methanol in a 1:1 ratio, supernatants were collected after centrifugation for 10 min at 10,000× *g*, filtered using 0.45 µm PTFE syringe filters and deposited into HPLC vials. The samples were separated with an Agilent HILIC-Z column (2.1 × 100 mm, 2.7 µM; Agilent) in an Agilent 1290 HPLC system coupled to a Q-Exactive Quadrupole Orbitrap MS. Mobile phases consisted of 20 mM ammonium formate in water (A) and 20 mM ammonium formate in 90% acetonitrile (B). Gradient conditions were as follows: 0 min, 100% B; 0.5 min, 100% B; 5.3 min, 80% B; 9.5 min, 30% B; 13.5 min, 30% B, 14.5 min 100% B and 16.5 min, 100% B. Heated electrospray ionization (HESI) was operated in positive mode at a capillary voltage of 3.4 kV. capillary temperature, 250  °C; sheath gas, 30.00 units; auxiliary gas, 8.00 units; probe heater temperature, 450  °C; S-Lens RF level, 60.00. Full MS scans were obtained in the mass range of *m*/*z* 58–870 at 35,000 resolution, MS/MS scans set at 17,500 resolution, isolation window of *m*/*z* 1.2, and collision energy of 28. 

### 4.4. Metabolite Identification 

The raw files were converted into .MZML format using ProteoWizard (Palo Alto, CA, USA) [43] and chromatogram alignment and deconvolution was completed using the XCMS package v1.36.0 in R [44]. Features were detected with the centWave method [45] at a 1 ppm tolerance and the prefilter was set to 3 to 5000, noise to 1000, and a signal-to-noise threshold of 5. The obiwarp [46] method was used to correct retention times. Features present in at least 25% of the samples were grouped. Two-thirds of the minimum value of each feature was used to replace zeros. The McMillan correction was utilized to remove large mass salt clusters and ionization artifacts [47]. Principal component analysis was completed using the FactoMineR package v. 2.3 in R [48] and the data was exported and analyzed. Score plots were generated using the R package ggplot2 v3.3.3 [42]. Biogenic amines were identified by exact mass and fragmentation patterns. 

### 4.5. Biogenic Amines Pathway Analysis

Amino acid sequences relevant to the biosynthetic and biocatalytic pathways of putrescine, cadaverine, and tyramine were downloaded (NCBI; May 2021). FASTA files containing the available *L. crispatus* coding sequences were analysed using blastp. A graphical summary indicating presence or absence of specific enzymes was elaborated using the R package ggplot2 v3.3.3 [43]. Pathways’ diagrams were plotted based on the information available at the Kyoto Encyclopedia of Genes and Genomes (KEGG) Pathway Database (San Francisco, CA, USA) [49,50,51].

### 4.6. Agar Well Diffusion Assays 

A series of agar well diffusion assays, a standard method used to detect bacteriocins and other antimicrobial compounds, were performed to identify the inhibitory profile of the strains [52,53]. *L. crispatus* strains were grown as a lawn across the surface for 48 h on Columbia blood agar (CBA) plates anaerobically in a BD GasPak™ EZ container systems at 37 °C (Franklin Lakes, NJ, USA). To obtain extracellular anti-microbial molecules, plates were then frozen at −80 °C for 1 h and thawed at room temperature for 1 h while inverted. Following thawing the resulting supernatant was collected from the lid of the plate, the volumes were normalized, the samples were neutralized using NaOH or HCl, and then all samples were filter sterilized (0.22 µm). To establish the inhibitory profile, indicator strains were plated on M17 agar (10% lactose). Next, wells of 1 cm in diameter were bored into the agar using the base of a 1000 µL pipette tip and 50 µL of each individual supernatant sample was deposited into the wells. Following an incubation period of 48 h at 37 °C the plates were imaged with a scale and the zones of inhibition were measured using ImageJ software [54]. 

Included in all assays were pH neutralized *E. faecalis* ATCC 19433, *L. helveticus*, and *P. pentosaceus*, *L. crispatus* supernatants, and a CBA supernatant brought to pH 5 using lactic acid as positive controls for bacteriocin production and a CBA supernatant sample that was neutralized, unaltered, and one that was brought to pH 5 with lactic acid and then neutralized as negative controls. The assays were repeated using indicator strains *E. faecium* ATCC 19434, *E. coli* UTI 89, *E. faecalis* ATCC 19433, *G. vaginalis* ATCC 14018, *P. bivia* ATCC 29303, and *C. albicans* TIMM 1768 (*n* = 4 biological replicates). 

To verify the presence of an inhibitory protein within the supernatants, an agar well diffusion assay with *E. faecium* ATCC 19,434 as an indicator strain was repeated in two parts with a small subset of strains, one with supernatants that were heated to 85 °C for 45 min prior to filter sterilization and a second where the supernatants were treated as described above. The remaining supernatants from this experiment were also subjected to treatment with trypsin, a protease, using 1 mg/mL final concentration and an agar well diffusion assay was conducted. 

### 4.7. Statistical Analysis 

Statistical analyses were completed using RStudio version 1.2.1335. Differences in inhibition areas between groups were evaluated using a linear mixed effects model to control for individual strain effects; the following R packages were used: rstatix v0.7.0 [55], emmeans v1.6.0 [56], and FSA v0.8.32 [57]. For differences of the amount of biogenic amines between health-status groups a T-test was performed using the R package ggsignif v0.6.1 [58]. Differences in biogenic amine amounts between strains were calculated using a one-way analysis of variance (ANOVA) with the Dunnett’s test, to correct heteroscedasticity marginal means were used and the matrix of co-variance was adjusted, this was performed using the R packages rstatix v0.7.0 [55], emmeans v1.6.0 [56], and sandwich v3.0-1 [59].

## Figures and Tables

**Table 1:** *L. crispatus* strains used in this study and the analyses performed on them. Strains were isolated from human vaginal swabs with *Lactobacillus*-dominated vaginal microbiota (LVM) or dysbiotic vaginal microbiota (DVM) [12].

Strain	Group	Metabolomics Analysis	Genomics Analysis	Antimicrobial Test	Strain	Group	Metabolomics Analysis	Genomics Analysis	Antimicrobial Test
RL02	DVM	yes	yes	yes	RL06	LVM	no	yes	no
RL03	LVM	yes	yes	yes	RL07	DVM	no	yes	no
RL05	LVM	yes	yes	yes	RL08	LVM	no	yes	no
RL09	LVM	yes	yes	yes	RL11	LVM	no	yes	no
RL10	LVM	yes	yes	yes	RL15	DVM	no	yes	no
RL13	DVM	yes	yes	yes	RL19	DVM	no	yes	no
RL14	DVM	yes	yes	yes	RL20	DVM	no	yes	no
RL16	LVM	yes	yes	yes	RL21	DVM	no	yes	no
RL17	DVM	yes	yes	yes	RL24	DVM	no	yes	no
RL23	DVM	yes	yes	yes	RL26	LVM	no	yes	no
RL25	DVM	yes	yes	yes	RL30	DVM	no	yes	no
RL27	LVM	yes	yes	yes	RL31	DVM	no	yes	no
RL28	DVM	yes	yes	yes	RL32	LVM	no	yes	no
RL29	LVM	yes	yes	yes	RL33	DVM	no	yes	yes
**Strains Added to Original Set**									
RL01	LVM	yes	no	yes					
RL04	LVM	yes	no	yes					
RL12	LVM	yes	no	yes					
RL22	LVM	yes	no	yes					

## Data Availability

Data will be available upon request.

## Supplementary Materials

Figure S1. PCoA of predicted functional capacity of strains based on mapping of genes to the EggNOG database. Figure S2. Zone of inhibition measurements (mm) from agar well diffusion assays featuring *L. crispatus* supernatants that were heated, treated with trypsin, and unheated against *E. faecium* indicator strain. Table S1. *L. crispatus* strains used in this study. Strains were isolated from human vaginal swabs with Lactobacillus-dominated vaginal microbiota (LVM) or dysbiotic vaginal microbiota (DVM). BioSampleDatabase accession numbers (NCBI: PRJNA390079) and bacterial collection strain numbers.

## References

[1] Petrova M.I., Lievens E., Malik S., Imholz N., Lebeer S. (2015). *Lactobacillus* species as biomarkers and agents that can promote various aspects of vaginal health. Front. Physiol..

[2] Barrons R., Tasone D. (2008). Use of *Lactobacillus* probiotics for bacterial genitourinary infections in women: A review. Clin. Ther..

[3] Schlabritz-Loutsevitch N., Gygax S.E., Dick E., Smith W.L., Snider C., Hubbard G., Ventolini G. (2016). Vaginal dysbiosis from an evolutionary perspective. Sci. Rep..

[4] McMillan A., Rulisa S., Sumarah M., Macklaim J.M., Renaud J., Bisanz J.E., Gloor G.B., Reid G. (2015). A multi-platform metabolomics approach identifies highly specific biomarkers of bacterial diversity in the vagina of pregnant and non-pregnant women. Sci. Rep..

[5] Reid G. (2018). Is bacterial vaginosis a disease?. Appl. Microbiol. Biotechnol..

[6] Cohen C.R., Wierzbicki M.R., French A.L., Morris S., Newmann S., Reno H., Green L., Miller S., Powell J., Parks T. (2020). Randomized trial of Lactin-V to prevent recurrence of bacterial vaginosis. N. Engl. J. Med..

[7] Andreu A., Stapleton A.E., Fennell C.L., Hillier S.L., Stamm W.E. (1995). Hemagglutination, adherence, and surface properties of vaginal *Lactobacillus* species. J. Infect. Dis..

[8] Kim J.-W., Rajagopal S.N. (2001). Antibacterial activities of *Lactobacillus crispatus* ATCC 33820 and *Lactobacillus gasseri* ATCC 33323. J. Microbiol..

[9] Nelson T.M., Borgogna J., Brotman R.M., Ravel J., Walk S.T., Yeoman C.J. (2015). Vaginal biogenic amines: Biomarkers of bacterial vaginosis or precursors to vaginal dysbiosis?. Front. Physiol..

[10] Puebla-Barragan S., Renaud J., Sumarah M., Reid G. (2020). Malodorous biogenic amines in *Escherichia coli*-caused urinary tract infections in women—A metabolomics approach. Sci. Rep..

[11] Borgogna J., Shardell M.D., Grace S.G., Santori E.K., Americus B., Li Z., Ulanov A., Forney L., Nelson T.M., Brotman R.M. (2021). Biogenic amines increase the odds of bacterial vaginosis and affect the growth of and lactic acid production by vaginal *Lactobacillus* spp.. Appl. Environ. Microbiol..

[12] van der Veer C., Hertzberger R.Y., Bruisten S.M., Tytgat H.L.P., Swanenburg J., de Kat Angelino-Bart A., Schuren F., Molenaar D., Reid G., de Vries H. (2019). Comparative genomics of human *Lactobacillus crispatus* isolates reveals genes for glycosylation and glycogen degradation: Implications for in vivo dominance of the vaginal microbiota. Microbiome.

[13] Pereira C.I., Matos D., San Romão M.V., Barreto Crespo M.T. (2009). Dual role for the tyrosine decarboxylation pathway in *Enterococcus faecium* E17: Response to an acid challenge and generation of a proton motive force. Appl. Environ. Microbiol..

[14] Gómez L.M., Sammel M.D., Appleby D.H., Elovitz M.A., Baldwin D.A., Jeffcoat M.K., Macones G.A., Parry S. (2010). Evidence of a gene-environment interaction that predisposes to spontaneous preterm birth: A role for asymptomatic bacterial vaginosis and DNA variants in genes that control the inflammatory response. Am. J. Obstet. Gynecol..

[15] Elli M., Zink R., Rytz A., Reniero R., Morelli L. (2000). Iron requirement of *Lactobacillus* spp. in completely chemically defined growth media. J. Appl. Microbiol..

[16] Pištěková H., Jančová P., Berčíková L., Buňka F., Sokolová I., Šopík T., Maršálková K., de Amaral O.M.R.P., Buňková L. (2020). Application of qPCR for multicopper oxidase gene (MCO) in biogenic amines degradation by *Lactobacillus casei*. Food Microbiol..

[17] Li B., Wang Y., Xue L., Lu S. (2021). Heterologous expression and application of multicopper oxidases from *Enterococcus* spp. for degradation of biogenic amines. Protein Pept. Lett..

[18] Borges S., Silva J., Teixeira P. (2014). The role of lactobacilli and probiotics in maintaining vaginal health. Arch. Gynecol. Obstet..

[19] Zamfir M., Callewaert R., Cornea P.C., Savu L., Vatafu I., De Vuyst L. (1999). Purification and characterization of a bacteriocin produced by *Lactobacillus acidophilus* IBB 801. J. Appl. Microbiol..

[20] McGroarty J.A., Reid G. (1988). Detection of a *Lactobacillus* substance that inhibits *Escherichia coli*. Can. J. Microbiol..

[21] Antonio M.A.D., Meyn L.A., Murray P.J., Busse B., Hillier S.L. (2009). Vaginal colonization by probiotic *Lactobacillus crispatus* CTV-05 is decreased by sexual activity and endogenous lactobacilli. J. Infect. Dis..

[22] Geshnizgani A.M., Onderdonk A.B. (1992). Defined medium simulating genital tract secretions for growth of vaginal microflora. J. Clin. Microbiol..

[23] Nilsen T., Nes I.F., Holo H. (2003). Enterolysin A, a cell wall-degrading bacteriocin from *Enterococcus faecalis* LMG 2333. Appl. Environ. Microbiol..

[24] Fremaux C., Klaenhammer T.R., De Vuyst L., Vandamme E. (1994). Helveticin J, a large heat-labile bacteriocin from *Lactobacillus helveticus*. Bacteriocins of Lactic Acid Bacteria.

[25] Jiang S., Cai L., Lv L., Li L. (2021). *Pediococcus pentosaceus*, a future additive or probiotic candidate. Microb. Cell Fact..

[26] Agudelo Higuita N.I., Huycke M.M., Gilmore M.S., Clewell D.B., Ike Y., Shankarn N. (2014). Enterococcal disease, epidemiology, and implications for treatment. Enterococci: From Commensals to Leading Causes of Drug Resistant Infection.

[27] Mysorekar I.U., Hultgren S.J. (2006). Mechanisms of uropathogenic *Escherichia coli* persistence and eradication from the urinary tract. Proc. Natl. Acad. Sci. USA.

[28] Anukam K.C., Reid G. (2008). Effects of metronidazole on growth of *Gardnerella vaginalis* ATCC 14018, probiotic *Lactobacillus rhamnosus* GR-1 and vaginal isolate *Lactobacillus plantarum* KCA. Microb. Ecol. Health Dis..

[29] Atassi F., Brassart D., Grob P., Graf F., Servin A.L. (2006). In vitro antibacterial activity of *Lactobacillus helveticus* strain KS300 against diarrhoeagenic, uropathogenic and vaginosis-associated bacteria. J. Appl. Microbiol..

[30] Panthee S., Hamamoto H., Ishijima S.A., Paudel A., Sekimizu K. (2018). Utilization of hybrid assembly approach to determine the genome of an opportunistic pathogenic fungus, *Candida albicans* TIMM 1768. Genome Biol. Evol..

[31] Gurevich A., Saveliev V., Vyanhi N., Tesler G. (2013). QUAST: Quality assessment tool for genome assemblies. Bioinformatics.

[32] Parks D.H., Imelfort M., Skennerton C.T., Hugenholtz P., Tyson G.W. (2015). CheckM: Assessing the quality of microbial genomes recovered from isolates, single cells, and metagenomes. Genome Res..

[33] Seemann T. (2014). Prokka: Rapid prokaryotic genome annotation. Bioinformatics.

[34] Page A.J., Cummins C.A., Hunt M., Wong V.K., Reuter S., Holden M.T.G., Fookes M., Falush D., Keane J.A., Parkhill J. (2015). Roary: Rapid large-scale prokaryote pan genome analysis. Bioinformatics.

[35] Stamatakis A. (2014). RAxML version 8: A tool for phylogenetic analysis and post-analysis of large phylogenies. Bioinformatics.

[36] Yu G., Smith D.K., Zhu H., Guan Y., Lam T.T. (2017). Ggtree: An R package for visualization and annotation of phylogenetic trees with their covariates and other associated data. Methods Ecol. Evol..

[37] Huerta-Cepas J., Szklarczyk D., Heller D., Hernández-Plaza A., Forslund S.K., Cook H., Mende D.R., Letunic I., Rattei T., Jensen L.J. (2019). eggNOG 5.0: A hierarchical, functionally and phylogenetically annotated orthology resource based on 5090 organisms and 2502 viruses. Nucleic Acids Res..

[38] Huerta-Cepas J., Forslund K., Coelho L.P., Szklarczyk D., Jensen L.J., von Mering C., Bork P. (2017). Fast genome-wide functional annotation through orthology assignment by eggNOG-mapper. Mol. Biol. Evol..

[39] Oksanen J., Blanchet G., Friendly M., Kindt R., Legendre P., McGlinn D., Minchin P., O’Hara R., Simpson G., Solymos P. (2020). Vegan: Community Ecology Package; R Package Version 2.5-7. https://cran.r-project.org/web/packages/vegan/vegan.pdf.

[40] Petrova M.I., Macklaim J.M., Wuyts S., Verhoeven T., Vanderleyden J., Gloor G.B., Lebeer S., Reid G. (2018). Comparative genomic and phenotypic analysis of the vaginal probiotic *Lactobacillus rhamnosus* GR-1. Front. Microbiol..

[41] Wuyts S., Wittouck S., De Boeck I., Allonsius C.N., Pasolli E., Segata N., Lebeer S. (2017). Large-scale phylogenomics of the *Lactobacillus casei* group highlights taxonomic inconsistencies and reveals novel clade-associated features. mSystems.

[42] Wickham H. (2016). ggplot2: Elegant Graphics for Data Analysis.

[43] Chambers M.C., Maclean B., Burke R., Amodei D., Ruderman D.L., Neumann S., Gatto L., Fischer B., Pratt B., Egertson J. (2012). A cross-platform toolkit for mass spectrometry and proteomics. Nat. Biotechnol..

[44] Smith C.A., Want E.J., O’Maille G., Abagyan R., Siuzdak G. (2006). XCMS: Processing mass spectrometry data for metabolite profiling using nonlinear peak alignment, matching, and identification. Anal. Chem..

[45] Tautenhahn R., Böttcher C., Neumann S. (2008). Highly sensitive feature detection for high resolution LC/MS. BMC Bioinform..

[46] Prince J.T., Marcotte E.M. (2006). Chromatographic alignment of ESI-LC-MS proteomics data sets by ordered bijective interpolated warping. Anal. Chem..

[47] McMillan A., Renaud J.B., Gloor G.B., Reid G., Sumarah M.W. (2016). Post-acquisition filtering of salt cluster artefacts for LC-MS based human metabolomic studies. J. Cheminform..

[48] Lê S., Josse J., Husson F. (2008). FactoMineR: An R package for multivariate analysis. J. Stat. Softw..

[49] Kanehisa M., Furumichi M., Sato Y., Ishiguro-Watanabe M., Tanabe M. (2021). KEGG: Integrating viruses and cellular organisms. Nucleic Acids Res..

[50] Kanehisa M. (2019). Toward understanding the origin and evolution of cellular organisms. Protein Sci..

[51] Kanehisa M. (2000). KEGG: Kyoto Encyclopedia of Genes and Genomes. Nucleic Acids Res..

[52] Holder I.A., Boyce S.T. (1994). Agar well diffusion assay testing of bacterial susceptibility to various antimicrobials in concentrations non-toxic for human cells in culture. Burns.

[53] Tahara T., Oshimura M., Umezawa C., Kanatani K. (1996). Isolation, partial characterization, and mode of action of Acidocin J1132, a two-component bacteriocin produced by *Lactobacillus acidophilus* JCM 1132. Appl. Environ. Microbiol..

[54] Schneider C.A., Rasband W.S., Eliceiri K.W. (2012). NIH Image to ImageJ: 25 years of image analysis. Nat. Methods.

[55] Kassambara A. (2021). Rstatix: Pipe-Friendly Framework for Basic Statistical Tests; R Package Version 0.7.0. https://cran.r-project.org/web/packages/rstatix/index.html.

[56] Lenth R. (2021). Emmeans: Estimated Marginal Means, Aka Least-Squares Means; R Package Version 1.6.0. https://cran.r-project.org/web/packages/emmeans/index.html.

[57] Ogle D., Wheeler P., Dinno A. (2021). FSA: Fisheries Stock Analysis; R Package Version 0.8.32. https://cran.r-project.org/web/packages/FSA/index.html.

[58] Ahlmann-Eltze C. (2021). Ggsignif: Significance Brackets for “Ggplot2”; R Package Version 0.6.1. https://cran.r-project.org/web/packages/ggsignif/ggsignif.pdf.

[59] Zeileis A. (2004). Econometric Computing with HC and HAC Covariance Matrix Estimators. J. Stat. Softw..

