# Supplementary material for: Interstrain Variability of Human Vaginal Lactobacillus crispatus for Metabolism of Biogenic Amines and Antimicrobial Activity against Urogenital Pathogens
Source: Molecules. 2021 Jul 27;26(15):4538. doi: 10.3390/molecules26154538 (PMC8347528; doi:10.3390/molecules26154538)
Supplement: Supplementary file 1 [file molecules-26-04538-s001.zip › molecules-1262872-supplementary.pdf]

Interstrain variability of human vaginal *Lactobacillus crispatus* for metabolism of biogenic amines and antimicrobial activity against urogenital pathogens

Scarlett Puebla-Barragan [1,2], Emiley Watson [1,2], Charlotte van der Veer [3], John A. Chmiel[1,2], Charles Carr[1],

Jeremy P. Burton[1,2], Mark Sumarah [4], Remco Kort[5,6],

and Gregor Reid [1,2]*

SUPPLEMENTARY MATERIAL

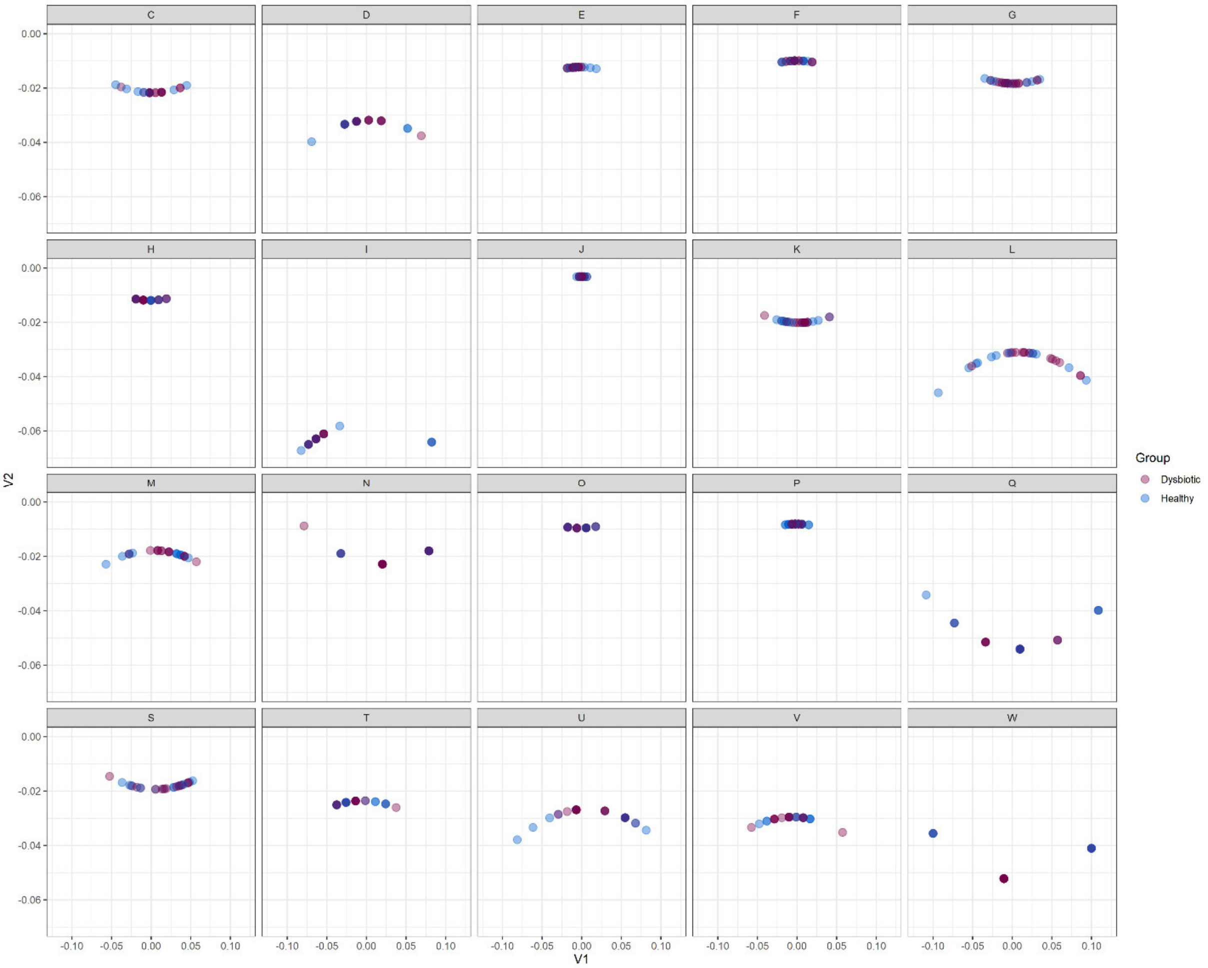

Figure S1. PCoA of predicted functional capacity of strains based on mapping of genes to the EggNOG database. Each letter represents a different functional category. [A] RNA processing and modification, [B] Chromatin structure and dynamics, [C] Energy production and conversion, [D] Cell cycle control, cell division, chromosome partitioning, [E] Amino acid transport and metabolism, [F] Nucleotide transport and metabolism, [G] Carbohydrate transport and metabolism, [H]

Coenzyme transport and metabolism, [I] Lipid transport and metabolism, [J] Translation, ribosomal structure and biogenesis, [K] Transcription, [L] Replication, recombination and repair, [M] Cell wall/membrane/envelope biogenesis, [N] Cell motility, [O] Post-translational modification, protein turnover, and chaperones, [P] Inorganic ion transport and metabolism, [Q] Secondary metabolites biosynthesis, transport, and catabolism, [R] General function prediction only, [S] Function unknown, [T] Signal transduction mechanisms, [U] Intracellular trafficking, secretion, and vesicular transport, [V] Defense mechanisms, and [W] Extracellular structures. Groups with <5 genes per strain were removed.

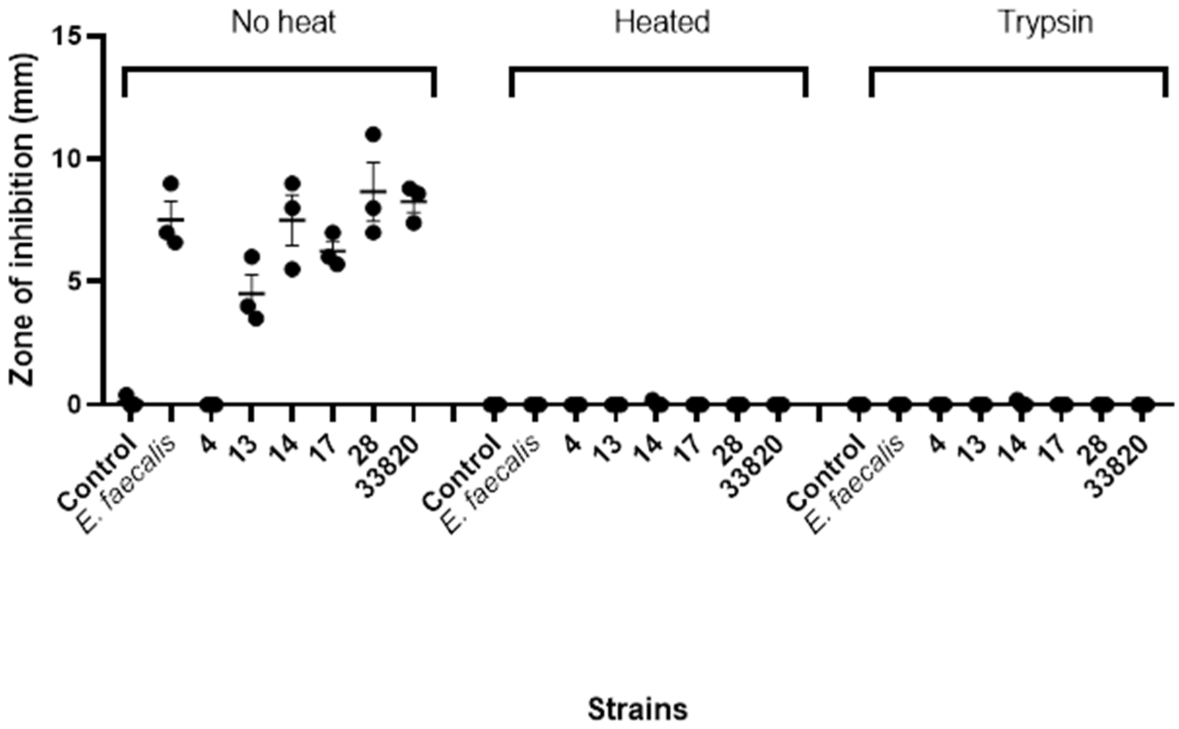

Figure S2. Zone of inhibition measurements (mm) from agar well diffusion assays featuring *L. crispatus* supernatants that were heated, treated with trypsin, and unheated against *E. faecium* indicator strain. Strains were grown for 24 hours on CBA plates and the individual supernatants were collected using the freeze/thaw method. *L. crispatus* supernatant volumes were then normalized, neutralized using HCl and NaOH, and filter sterilized. Additional CBA plates as controls without bacterial growth, were subjected to the same incubation and collection method. 1/3 each supernatant was heated to 85°C for 45 minutes, and 1/3 of the supernatants were treated with 1mg/mL of trypsin. Included in the controls was a sample that was neutralized, one that was left at its original pH, one that was brought to a pH of 5 using lactic acid and then neutralized using NaOH, and one that was brought to a pH of 5. All controls were then filter sterilized. 250µl of the indicator strain was plated on m17 agar, 1cm holes were bored, and 50 µl of each supernatant was deposited into each well. Following incubation at 37°C for 48 hours the zones of inhibition were measured (N=4).

Table S1. *L. crispatus* strains used in this study. Strains were isolated from human vaginal swabs with *Lactobacillus*-dominated vaginal microbiota (LVM) or dysbiotic vaginal microbiota (DVM) [1]. BioSample Database accession numbers (NCBI: PRJNA390079) and bacterial collection strain numbers.

Strain	BioSample Database DDBJ/ENA/GenBank	Collection	Strain	BioSample Database DDBJ/ENA/GenBank	Collection
**RL02**	SAMN07213039	NCCB 100711	**RL21**	SAMN07213201	NCCB 100723
**RL03**	SAMN07213108	not available	**RL22**	not available	not available
**RL05**	SAMN07213187	not available	**RL23**	SAMN07213203	NCCB 100724
**RL06**	SAMN07213188	not available	**RL24**	SAMN07213204	NCCB 100725
**RL07**	SAMN07213189	NCCB 100713	**RL25**	SAMN07213205	NCCB 100726
**RL08**	SAMN07213190	not available	**RL26**	SAMN07213206	not available
**RL09**	SAMN07213191	NCCB 100714	**RL27**	SAMN07213207	NCCB 100727
**RL10**	SAMN07213192	NCCB 100715	**RL28**	SAMN07213208	NCCB 100728
**RL11**	SAMN07213193	not available	**RL29**	SAMN07213209	not available
**RL13**	SAMN07213194	NCCB 100716	**RL30**	SAMN07213210	NCBB 100730
**RL14**	SAMN07213195	NCCB 100717	**RL31**	SAMN07213211	not available
**RL15**	SAMN07213196	NCCB 100718	**RL32**	SAMN07213212	not available
**RL16**	SAMN07213197	NCCB 100719	**RL33**	SAMN07213213	NCCB 100731
**RL17**	SAMN07213198	NCCB 100720			
**RL19**	SAMN07213199	NCCB 100721	**RL01**	not available	not available
**RL20**	SAMN07213200	NCCB 100722	**RL04**	Not available	Not available
			**RL12**	not available	not available
			**RL22**	not available	not available

Reference

1. van der Veer, C.; Hertzberger, R.Y.; Bruisten, S.M.; Tytgat, H.L.P.; Swanenburg, J.; de Kat Angelino-Bart, A.; Schuren, F.; Molenaar, D.; Reid, G.; de Vries, H.; Kort, R. Comparative genomics of human *Lactobacillus crispatus* isolates reveals genes for glycosylation and glycogen degradation: implications for *in vivo* dominance of the vaginal microbiota. *Microbiome* **2019**, *7*, 49.
